# Nitric oxide and cancer: a review

**DOI:** 10.1186/1477-7819-11-118

**Published:** 2013-05-30

**Authors:** Sheetal Korde Choudhari, Minal Chaudhary, Sachin Bagde, Amol R Gadbail, Vaishali Joshi

**Affiliations:** 1 Department of Oral Pathology & Microbiology, Yerala Dental College and Hospital, Institutional Area, Sector 4, Kharghar, Navi Mumbai, Maharashtra, 410 210, India; 2 Department of Oral Pathology & Microbiology, Sharad Pawar Dental College, DMIMS, Sawangi(M), Wardha, Maharashatra, 442 004, India; 3 Department of Oral Surgery, Yerala Dental College and Hospital, Institutional Area, Sector 4, Kharghar, Mumbai, Maharashtra, 410 210, India

**Keywords:** Breast cancer, Gastric cancer, Lung cancer, Head and Neck cancer, H. Pylori, Human papillomavirus, Nitric oxide, Nitric oxide synthase

## Abstract

Nitric oxide (NO), is a ubiquitous, water soluble, free radical gas, which plays key role in various physiological as well as pathological processes. Over past decades, NO has emerged as a molecule of interest in carcinogenesis and tumor growth progression. However, there is considerable controversy and confusion in understanding its role in cancer biology. It is said to have both tumoricidal as well as tumor promoting effects which depend on its timing, location, and concentration. NO has been suggested to modulate different cancer-related events including angiogenesis, apoptosis, cell cycle, invasion, and metastasis. On the other hand, it is also emerging as a potential anti-oncogenic agent. Strategies for manipulating *in vivo* production and exogenous delivery of this molecule for therapeutic gain are being investigated. However, further validation and experimental/clinical trials are required for development of novel strategies based on NO for cancer treatment and prevention. This review discusses the range of actions of NO in cancer by performing an online MEDLINE search using relevant search terms and a review of the literature. Various mechanisms by which NO acts in different cancers such as breast, cervical, gastric,colorectal, and head and neck cancers are addressed. It also offers an insight into the dichotomous nature of NO and discusses its novel therapeutic applications for cancer prevention and treatment.

## Review

### Introduction

Nitric oxide (NO) is a short-lived, endogenously produced gas that acts as a signaling molecule in the body. Ignarro et al. and Palmer et al. simultaneously identified NO as the endothelium-derived relaxing factor in 1987 [1,2]. It is synthesized by nitric oxide synthase (NOS) enzymes; produced by mammalian cells at an appropriate magnitude and tempo, it serves as a key signaling molecule in various physiological processes. On the other hand, excessive and unregulated NO synthesis has been implicated as causal or contributing to pathophysiological conditions including cancer. Expression of NOS has been detected in various cancers such as cervical, breast, central nervous system, laryngeal, and head and neck cancers [3-7]. NO has been suggested to modulate different cancer-related events [8]. However, several lines of research have indicated that NO may have dual effects in cancer. At concentrations measurable in many different types of clinical samples, NO seems to promote tumor growth and proliferation. In contrast to this, NO is said to have tumoricidal effects;various direct and indirect mechanisms have been proposed for its antitumor properties [9,10], although there is lack of data directly on cancer patients. Nevertheless, the tumoricidal properties of NO are being investigated for therapeutic purposes. NO is used alone or in combination with other cytotoxic agents. In order to obtain a better insight into the dichotomous nature of NO, an online search using proper search terms through MEDLINE was undertaken and the relevant literature was reviewed. This review discusses the diverse actions of NO in cancer and NO’s novel applications in cancer treatment and prevention.

### Biological and physiological aspects of NO

NO, a short-lived endogenously produced gas, is synthesized by a complex family of NOS enzymes. Mammalian cells are endowed with three genes encoding distinct isoforms of NOS– NOS1, NOS2, and NOS3 –with 51-57% homology between isoforms and different localizations, regulation, catalytic properties, and inhibitor sensitivity. NOS1, also known as nNOS (isoform first purified and cloned from neuronal tissue), and NOS3 or eNOS (isoform first found in endothelial cells) are also termed as *constitutive* since they are expressed continuously in neurons and endothelial cells, respectively. They are also dependent on a rise in tissue calcium concentration for activity and therefore produce low, transient concentrations of NO. In contrast, NOS2 is an inducible, calcium-independent isoform, also called iNOS. Unlike NOS1 and NOS3, induction of NOS2 results in continuous production of NO [11]. It is inducible by immunological stimuli in virtually all nucleated mammalian cells. Once induced, the enzyme continues to produce much higher NO concentrations for many hours or even days. An important regulator of NOS2 is the tumor suppressor gene p53 which senses raised cellular NO and inhibits NOS2 by a negative feedback loop [12]. This relationship has important implications in cancer.

Contrary to conventional biosignaling molecules that act by binding to specific receptor molecules, NO manifests its biological actions via a wide range of chemical reactions. The precise reactions depend on the concentration of NO achieved and on subtle variations in the composition of intra- and extracellular milieu [11]. Under normal physiological conditions, cells produce small but significant amounts of NO which contribute to regulation of anti-inflammatory effects and its antioxidant properties [13,14]. However, in tissues with a high-output of NO, iNOSisupregulated and effects such as nitration (addition of NO_2_), nitrosation (addition of NO^+^), and oxidation will prevail [13]. Interaction of NO with O_2_ or O_2_^-^ results in formation of reactive nitrogen species (RNS). The RNS, dinitrogen trioxide (N_2_O_3_) and peroxynitrite (ONOO), can induce two types of chemical stresses,nitrosative and oxidative [15]. N_2_O_3_ is a potent nitrosating agent which has been shown to N- and S- nitrosate a variety of biological targets to yield potentially carcinogenic nitrosamines and nitrosothiol derivatives. N-nitrosation may have important implications in the known association between chronic inflammation and malignant transformation [15,16]. O_2_^-^ and NO may rapidly interact to produce the potent cytotoxic oxidants peroxynitrite (ONOO^-^) and its conjugate acid ONOOH. Peroxynitrite in natural solution is a powerful oxidant, oxidizing thiols or thioethers, nitrating tyrosine residues, nitrating and oxidizing guanosine, degrading carbohydrates, initiating lipid peroxidation and cleaving DNA, which has important implications in cancer [17,18].

### Diverse actions of NO in cancer

NO has been reported to exert dichotomous effects within the multistage model of cancer (Tables 1 and 2). It modulates different cancer-related events including angiogenesis, apoptosis, cell cycle, invasion, and metastasis [8] (Table 1). In contrast to tumor promoting effects, NO has also been reported to have tumoricidal effects (Table 2). Understanding its role in tumor biology will help in reducing the controversy and confusion and will help in developing novel NO based therapies which will prove helpful in preventing and treating various human cancers.

**Table 1:** Diverse actions of NO in cancer: tumor promoting role of NO

**Tumor promoting role**	
**Role of nitric oxide (NO)**	**Mechanism by which NO acts**
Genotoxicmechanisms	Formation of toxic and mutagenic species
	Direct modification of DNA– strand breaks, oxidation and deamination of nucleic acids
	Inhibition of systems required to repair DNA lesions
Antiapoptotic effects	GC to AT mutations in p53– loss of its repressor activity
	Direct inhibition of caspase activity through s-nitrosylation of the cysteine thiol
	Inhibition of cytochrome C release
	Increase in Bcl-2 expression
	Suppression of ceramide generation
	Activation of cyclooxygenase
Induces and promotes angiogenesis (iNOSandeNOS)	Dilatation of arteriolar vessels by eNOS
	VEGF release and NO stimulation of hyperpermeability of vascular endothelium
	Increased production of PGE2 resulting in increased tumor vasculature permeability
	Activation of COX-2 which stimulates production of proangiogenic factors and prostaglandins
Limits host immune response against tumor	Suppression of proliferation and infiltration of leukocytes
	Low leukocyte-endothelial interaction
Promotes metastasis	Promotes lymphangiogenesis and spread to lymph nodes possibly through involvement of VEGF-C
	Upregulation of MMP-2 and −9
	Downregulation of TIMP-2 and-3

**Table 2:** Diverse actions of NO in cancer: tumoricidal role of NO

**Tumoricidal role**	
**Role of NO**	**Mechanism by which NO acts**
Cytostatic and\or cytotoxic effect on tumor cells	NO suppresses cellular respiration and also shifts iron metabolism
	Suppression of DNA synthesis
	Proapoptotic modulator by activating caspase family proteases, upregulation of p53, alteration in expression of apoptosis-associated proteins

**Figure 1:**
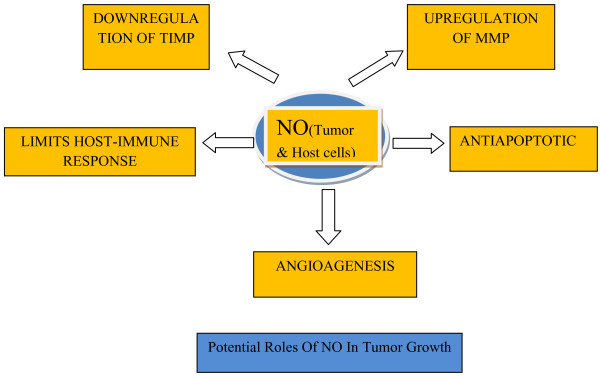
Potential roles of NO in tumor growth.

The effects of NO in tumor biology are broad, spanning its involvement in cellular transformation, formation of neoplastic lesions, and initiation and regulation of the metastatic cascade. NO participates in genotoxic events [19,20]. It may mediate DNA lesions by formation of toxic and mutagenic species, by direct modification of DNA, or by inhibition of DNA repair mechanisms [21]. RNS can mediate DNA strand breaks and can also yield different types of mutations in DNA [22,23]. NO production via iNOS may directly induce GC to AT mutations in p53 which may contribute to loss of its repressor activity. NO directly inhibits activity of caspases providing an efficient means to block apoptosis. Other antiapoptotic effects of NO rely on NO/cGMP dependent inhibition of cytochrome C release, increase in Bcl-2 expression that controls the mitochondrial permeability transition pore, induction of heat-shock protein (Hsp) 70 and Hsp 32, suppression of ceramide generation [24], and activation of cyclooxygenase-2 [24,25]. NO plays an important role in tumor progression by regulation of angiogenesis. Endogenous NO promotes tumor blood flow via dilatation of arteriolar vessels. It decreases leukocyte endothelial adhesive interactions and increases vascular permeability [26]. Studies have shown that VEGF released as a purified protein or produced by tumor cells requires a functional NO/cGMP pathway within the end compartment to promote neovascular growth. Another mechanism by which NO promotes angiogenesis is by activation of COX-2 which stimulates the production of proangiogenic factors and prostaglandins. NO also has an invasion stimulating effect which is mediated by upregulation of MMP-2 and MMP-9 (matrix metalloproteinases), and downregulation of TIMP-2 and possibly TIMP-3 (tissue inhibitors of MMP) [27]. Studies have indicated that NO limits leukocyte cell proliferation which has adverse consequences on the antitumor response of the host [23]. In this way NO may be involved in the growth and spread of tumors (Figure 1).

### NO in various human cancers

Nitric oxide seems to play diverse roles in various human cancers [3-7]. Understanding different actions of NO in these cancers at the molecular level can help in providing NO based diagnostic or prognostic markers and also in devising potential strategies for prevention and treatment of these cancers.

### NO and breast cancer

Breast cancer is currently the most common cancer in women worldwide, both in developed and developing countries [28]. NOhas been investigatedregarding its possible involvement in the promotion of breast carcinoma. Specific expression of NOS has been reported in breast cancer tissues [29] and in breast carcinoma cell lines [30]. Increased amounts of NO have been observed in blood of breast cancer patients [31] and higher NOS activity has been found in invasive breast tumors when compared with benign or normal breast tissue [29]. Authors have found a high rate of NOS in *in situ*carcinoma [29]. Furthermore, NOS activity has been found to be higher in advanced grades of breast carcinomas [31]. All these findings suggest that NOS expression in breast cancer may be an early event in carcinogenesis.

NO is reported to have several important effects in the control of neoplasms. Jadeskiet al. suggested that the presence of eNOS in breast apocrine metaplastic cells of fibrocystic disease in the human may promote the progression of metaplastic epithelium into carcinoma [32]. NO increases tumor blood flow and promotes angiogenesis, which could explain the positive correlation between NO biosynthesis and grade of malignancy [31]. Nitrotyrosine, a biomarker of NO, was found to be correlated with VEGF-C expression and lymph node metastasis in breast cancer suggesting the role of NO in progression of breast carcinoma [33]. Switzer et al. showed that NOS2 expression in human breast tumors is functionally linked to poor patient survival [34].

### NO and hormones in breast cancer

Unlike other types of cancer, tumors of the breast are greatly influenced by steroid hormones. Recent findings implicate NO pathway in some of their effects. Estrogen and progesteronecan regulate NOS and, in turn, the NO produced has profound consequences on tumor cell homeostasis [30,35]. It has been found that estrogen stimulates eNOS release in breast tissue [36] where it acts as a free radical. eNOS expression has been found to be strongly correlated with estrogen receptor expression in a human breast cancer cell line, suggesting free radicals as possible causes of breast cancer [36]. Progesterone has been found to activate iNOS expression. It has been suggested that the low levels of NO produced by eNOS mediate the proliferative effect of estrogen [37]. On the other hand, an increase in apoptosis in response to progesterone could implicate high levels of NO produced by induction of iNOS expression [37]. Thus an understanding of the mechanisms and interactions of steroid hormones with the NO pathway could lead to the development of new approaches and strategies for the effective treatment of breast cancer.

### NO and cervical cancer

Cervical cancer is the second most common cancer in women worldwide, with >200,000 deaths annually [38]. Epidemiological studies have revealed a number of risk factors, including smoking, multiparity, long-term use of oral contraceptives, chronic inflammation, and other sexually transmitted infections (e.g., chlamydia trachomatis and herpes simplex virus type 2) [39]. Interestingly, these cofactors all increaseNO levels in the cervical microenvironment [40-42]. Significantly higher levels of NO were observed in serum of patients with cervical cancer as compared to healthy controls [43,44]. Increased NO levels and markers of NO-mediated mutagenesis have been observed in the cervixes of women with cervical intraepithelial neoplasia [45,46]. All these findings suggest that NO has potential mutagenic and carcinogenic activity in cervical cancer.

#### Human papillomaviruses, NO and cervical cancer

Infections with any of the 15 types of human papillomaviruses (HPV) cause virtually all cases of cervical and other anogenital cancers [47]. According to Wei et al., NO acts as a molecular cofactor with HPV infection in cervical carcinogenesis [48]. Wei et al. found that the presence of HR-HPV is associated with an increased release of NO in the human uterine cervix [49] and that physiological doses of NO could promote malignant progression of HPV-infected cells *in vivo*. According to them, various epidemiologically defined cofactors for cervical cancer increase NO levels in the cervical microenvironment [48]. This increase in NO causes earlier mRNA expression, decreased pRb and p53 levels, low p53 activity, and apoptotic indices in HPV-infected cells in the cervix, resulting in increased survival of mutant cells leading to carcinogenesis [48].

### NO and lung cancer

Tobacco smoke is the main cause for lung cancer [50,51]. It leads to chronic airway inflammation with accumulation and activation of leukocytes which produce high levels of ROS and NO [52]. It has been demonstrated that NO, nitrite, and nitrotyrosine are increased in patients with lung cancer [49]. Chen et al. found significantly higher levels of iNOS/NO in lung cancer tissues of smokers than that of non-smokers [53]. Strong immunoreactivity for iNOS and eNOS was observed in dysplastic lesions in the lung [54]. Certain hexavalent chromium [Cr(VI)] compounds have been postulated to play a significant role in pulmonary tumorigenesis. Forbes et al.concluded that repetitive exposure to particulate chromate induces an inflammatory environment in the lung, accompanied by enhanced NO production, which may promote lung carcinogenesis [55].

NO may contribute to lung carcinogenesis by nitration of proteins. NO and its metabolites interact with ROS to generate potent nitrating agents leading to 3-nitrotyrosine formation in proteins [56,57], which is one of several chemical modifications that occur during oxidative/nitrosative stress [57,58]. At high levels, NO inactivates p53 [59] and p53 inactivation by nitration could also contribute to carcinogenesis given that over 90% of lung tumors are with defective p53 [52]. With broad effects on angiogenesis, glycolysis, p53 activity, antioxidant potential in the lung, and alteration of cell growth pathways, NO may create a microenvironment that promotestumorigenesis and/or promotes tumor heterogeneity leading to metastasis [52]. The prognosis of lung cancer is still poor because of the absence of valid approaches to its early detection. Exhaled breath analysis and exhaled NO measurements may provide useful assays in predicting diagnosis and disease progression [60].

### NO in gastric cancer

Despite advances in surgical treatment and chemotherapy, gastric cancer remains a major global health burden; various etiologic factors have been linked with the disease. It is widely accepted that *H. pylori* infection and high salt intake are positively associated with this neoplastic process. Controversial associations have been found with smoking or drinking habits [61]. The three enzymatic sources of NO, nNOS, eNOS, and iNOS, have been characterized in the gastrointestinal tract [62]. There is enhanced expression of iNOS and eNOS in human colorectal cancers [63]. Colon cancer tissue has also been found to express NOS mRNA [64].

Gastric carcinogenesis (GC) is considered as a multistage progressive process. The early indicator for GC predisposition is abnormal hyperproliferation of gastric epithelial cells, such as chronic atrophic gastritis (CAG), dysplasia (DYS) and intestinal metaplasia (IM), which have been considered as precancerous lesions of GC [65,66]. In a study by Feng et al., when the lesions progressed from normal to chronic superficial gastritis(CSG),CAG, IM, DYS, and finally to GC, the positive immunostaining rates for p53, iNOS, and VEGF were found to be significantly increased [64]. The study demonstrated that the positive immunostaining rates of iNOS were correlated well with GC lymph node metastasis. All these findings suggested a role of NO in the initiation and progression of GC. NOS can also deaminate DNA and cause mutations of tumor suppressor genes, and possibly other oncogenes, such as c-met, and initiate genetic alterations of gastric cells leading to gastric malignancy [23].

#### H.pylori, NO and gastric cancer

GC may be considered the result of interplay between host genetic profile and environmental toxic agents [67]. The link between *H. pylori* infection and GC has been demonstrated by epidemiological data and in experimental animal models [68-71]. Overproduction of NO via inducible iNOS is suggested to be a significant pathogenic factor in *H. pylori*-induced gastritis [72]. Exposure of gastric epithelial cells to bacterium results in the generation of reactive oxygen species (ROS) and iNOS that, in turn, may cause genetic alterations leading to GC in a subset of subjects [67].

An increasing frequency of p53 abnormalities occurs as the gastric mucosa progresses from gastritis, through IM, DYS, and early to advanced invasive GC [73]. C to T mutations in p53 are induced by NO [74,75] which might have been produced during *H. pylori* infections. Based on these facts, it can be said that *H. pylori* may lead to GC through overproduction of NO, as one of the mechanisms.

### NO in brain tumors

NO influences a great variety of vital functions including vascular tone and neurotransmission. NO emerges as an important mediator of neurotoxicity in a variety of disorders of the central nervous system (CNS). nNOS expression may act as a putative useful indicator of brain tumor differentiation and malignancy [75]. Cobbs et al. examined human brain tumors for three NOS isoforms and NADPH diaphorase, a histochemical marker of NOS activity in the brain. Data of their study suggested that malignant central nervous system neoplasms express unexpectedly high levels of NOS and suggest that NO production may be associated with pathophysiological processes important to these tumors [76].

### NO in head and neck cancer

Oral squamous cell carcinoma (OSCC) is the sixth most common malignancy and a major cause of morbidity and mortality [77]. The high incidence of oral cancer and oral pre-cancer has been linked with habits of tobacco chewing and smoking [78,79]. It is reasonable to assume that components of tobacco, as initiators of inflammatory response, could be responsible for the generation of ROS/RNS that may lead to lipid peroxidation, enhanced NO products, and deranged antioxidant defense system in tobacco users [80]. The damage to genes sustained by elevated ROS/RNS could be one of the mechanisms by which cancer arises in long-term tobacco abuse [80]. Raised levels of NO_2_ and NO_3_ were noted in patients with oral pre-cancer [81,82] and in healthy individuals with tobacco habit [80,81]. This indicates potential of nitrosative injury in tobacco users and,therefore, NO may have clinical relevance as a biomarker of inflammation and estimation of cancer risk in pre-cancer patients or in healthy tobacco users.

Alcohol intake is related to an increased predisposition to oral cancer [83]. Cooper and Magwere suggested that stimulation of NO production by ethanol is likely to play an important role in the etiology of some cancers, including head and neck cancer, which preferentially rely on NO signaling [84]. Taken together, these facts implicate the role of NO in development of oral cancer. Very few studies have evaluated the role of NO in oral pre-cancer. Whether NO actually acts as a protumoral agent at a concentration which is present in oral pre-cancer needs further evaluation. Studies are required to know the exact role of NO in oral pre-cancer which will be helpful in intervening the cancer process.

#### HPV, NO and head and neck cancer

The steady increase in the incidence of oropharyngeal cancers over the last four decades has been mainly attributed to oral HPV infection, which has been accepted as an etiological factor for a subset of head and neck squamous cell carcinoma (HNSCC) [85-88]. HPV-positive HNSCCs have a unique risk factor profile. These tumors are more common in younger patients, have a male predominance, and are often staged higher, yet have a survival advantage. These patients are less likely to have used tobacco and alcohol excessively [89]. An association between chronic inflammation such as through HPV infection and oral cancer is biologically plausible. It is known that chronic inflammation can lead to the production of NO which in turn can mediate DNA damage. Considering these facts, the role of NO in HPV associated head and neck cancer in patients without habits, needs an evaluation.

### Tumoricidal *versus* tumor promoting effect of NO

Although several reports have addressed the protumoraleffects of NO, as mentioned above, few have demonstrated the contrasting role of NO in mediating tumor regression [9,90,91]. It has been reported that NO derived from macrophages, Kupffer cells, natural killer cells, and endothelial cells participates in tumoricidal activity against many types of tumors [90,92]. These studies suggest that NO has a cytostatic and/or cytotoxic effect on tumor cells (Table 2, Figure 1). Several molecular targets, such as aconitase and ribonucleotidereductase, have been implicated in the cytostatic/cytotoxicity effects mediated by NO. NO has been proposed to cause suppression of DNA synthesis through the salvage pathway [93]. Long standing overproduction of NO acts as a proapoptotic modulator, activating caspase family proteases through the release of mitochondrial cytochrome C into the cytosol, upregulation of p53 expression, and alterations in the expression of apoptosis-associated proteins including the Bcl-2 family [24]. A high NO level has been proposed to suppress metastasis [94]. Baritakiet al. have shown that high levels of NO derived from the NO donor DETA-NONOate inhibits epithelial-mesenchymal transition (EMT) and reverses both the mesenchymal phenotype and the invasive properties of human prostate metastatic cells [95]. Findings of the study by Bonavida et al. have also suggested that NO donors may prove to be potential therapeutic agents in both reversal of drug resistance and the inhibition of EMT and metastasis [96]. Although these tumoricidal roles of NO have been proposed, most experiments have been performed *in vitro*[11,97] and such findings have not been reported in cancer patients. It has been suggested that NO concentrations found in OSCC and other solid tumors are insufficient to produce apoptosis [98] and other tumoricidal effect and are likely to facilitate angiogenesis and tumor dissemination [99]. Further, whether NO has an inhibitory or stimulatory effect on the cancer process initially depends on the concentration of NO achieved and also on other factors such as the type of cell exposed, the redox state, final intracellular concentration, duration of exposure, etc., in the tumor bed. Once the cancer has begun, NO seems to play a protumoral role rather than antitumoralone as the concentration required to cause tumor cell cytotoxicity cannot be achieved by cancer cells [100]. However, considering the cytostatic and/or cytotoxic effects of NO, strategies are being developed to manipulate NO levels in the tumor environment for therapeutic gain.

### NO as a novel cancer therapeutic

NO may exert a biphasic response, such that when NO levels go beyond a critical concentration that would be suitable for tumor growth and survival, growth arrest and/or apoptotic pathways are initiated. These characteristics of NO have been exploited therapeutically with impressive effects in pre-clinical models of cancer to slow tumor growth and to enhance the efficacy of both chemotherapy and radiotherapy [101]. Researchers are investigating various strategies for manipulating *in vivo* production and exogenous delivery of this molecule, includingiNOS gene therapy, iNOS induction, and administration of NO donor drugs [94] for therapeutic gain.

Transfer of NOS-encoding cDNA sequences into cancer cells for gene therapy purposes was thought to be one of the mechanisms for delivery of NO. However, as both retroviral and adenoviral vectors may be hazardous to the host, cell-based approaches to overcome the problems associated with gene therapy [102] are being sought. Further work into the precise mechanisms of this process is required.

Alternative mechanisms for NO delivery would be the use of NO releasing drugs or NO donors. These are capable of causing sustained release of NO with a wide range of half-lives, and with predictable estimated doses. They can simultaneously exert a multitude of anticancer activities including enhancement of apoptotic stimuli, inhibition of metastasis, inhibition of angiogenesis, and inhibition of hypoxia, depending on concentration of NO donor and on the cancer type and stage [103].

Several promising findings strongly support the therapeutic application of NO donors in cancer treatment, used alone or in combination with other subtoxic doses of cytotoxic agents. NO donors have been shown to have the dual function of both sensitizing tumor cells to chemotherapy and immunotherapy and of being involved in the regulation and inhibition of metastasis [104]. NO donors belonging to the class of diazeniumdiolates are promising as they have been shown to be effective chemo- and radio-sensitizing agents along with other attractive properties such as long half-lives and target tissue specific delivery. The role of nitro-glycerine as a chemo-sensitizing agent as demonstrated by Yasuda et al. [105,106], promises a safe and affordable alternative for the management of resistant or metastatic tumors. According to Bonavida and Baritaki [107], NO donors may be considered as novel potential therapeutic agents with dual roles in the treatment of patients with refractory cancer and in the prevention of the initiation of the metastatic cascade via EMT.

However, the therapeutic application of NO donors has been limited by potential systemic effects exerted *in vivo*. These adverse effects include vasodilation leading to pronounced hypotension and accumulation of toxic metabolites such as cyanide [108]. The need to develop the ideal NO donor with maximal anti-proliferative properties and minimal side effects has led to the invention of NO-hybrids. NO-hybrids are providing a unique niche in the armamentarium of anticancer agents. Combining NO to existing drugs affords an advantage of adding or potentiating the effects of NO to the benefits of drugs like NSAIDs or statins. NO-drug hybrids such as NO-NSAIDs demonstrate promise as anti-cancer agents and are in clinical trials by NCI-sponsored phase I randomized studies (i.e., NO-aspirin in high-risk patients with colorectal cancers) [109].

The synthesis of molecules capable of releasing optimal amounts of NO at the right time and the right place poses a great challenge to pharmaceutical research. NO donors can be incorporated into or chemically linked to biopolymers, mimicking endogenous NO production at a target site [110,111]. Nanomaterials are currently being harnessed to load high amounts of NO; they are quite stable, are sometimes photoactive, and possess demonstrable biological activity. Their surfaces can also be chemically modified and optimized for specific medical applications. They may facilitate the development of systems for simultaneous therapeutic and diagnostic applications [112]. These nanoparticles can be prepared by physiochemical, chemical, and mechanical methods [113]. However, drug release from particles may vary according to the polymer used or the drug encapsulated [114]. Nanocarriers of NO make the agent more available to systemic circulation and can also enhance the NO target [112].

A small number of reports have been published on the topical delivery of NO using polymeric systems. Kanayama et al. [115] reported that PEGylated polymer micelles may be capable of delivering exogenous NO to tumor cells in a photocontrolled manner, resulting in an NO-mediated antitumor effect, which indicates the promise of this polymeric system in NO-based tumor therapy. Friedman et al. reported that therapeutic levels of NO, in controlled and sustained manner, can be achieved by using combination of glassy matrices and hydrogels [116]. NO-releasing hydrogel/glass hybrid nanoparticles are preferable to other NO-releasing compounds as they depend only on rate of hydration and not on chemical decomposition or enzymatic catalysis [117]. [Ru(Terpy)(bdqi)NO](PF6)3, a NO donor nitrosyl ruthenium complex, has been bound to lipid nanocarriers for topical administration. This system exhibited improved stability in the skin and NO release by visible light irradiation, with potential applications in the treatment of skin cancer [118]. Stevens et al. engineered NO-releasing SiNPs for NO delivery to human ovarian cancer cells for their inhibition [119]. Another class of liposomes that can be successfully used as nanocarriers are thermosensitive liposomes; they can be employed in the storage, delivery, and active release of NO in a heat-mediated manner [120]. These thermo-sensitive liposomes containing NO may have applications in anticancer therapeutics as heat is generated in tumor tissue [121].

Fluorescent nanocrystals, also known as quantum dots (QDs) can be linked to NO-donor molecules. These can specifically lead to effective treatment of large tumors by photodynamic therapy [116-122]. In this case, the nitrosyl compounds can generate, under light application, ROS and NOS via QD excitation, enabling tumor cell death [117-124]. The preliminary *in vitro* experiments with neuroblastoma cells have demonstrated that the combination of nano-delivery and chemotherapy enhances antitumor activity of chemotherapeutics [125]. Current nanotechnology-based systems are highly promising but there are currently no commercially available nano- or microcarriers for NO delivery. Giles et al. have recently reported the development of two photolabileNO-releasing prodrugs, tert-butyl S-nitrosothiol and tert-dodecane S-nitrosothiol. They confirmed that irradiation induced highly significant increase in cytotoxicity in A549 lung carcinoma cells by these drugs. These prodrugs can be further explored to have applications in chemical biology studies and chemotherapy [126]. Thus NO appears to be a potentially promising agent for the treatment of cancer and prevention of metastatic cascade and therefore further studies are required to clearly understand the complex and wide-ranging roles of NO in order to facilitate its therapeutic use.

## Conclusion

NO is a relatively stable, free radical gas that readily diffuses into cells and cell membranes where it reacts with molecular targets. The precise reactions of NO depend on the concentration of NO achieved and on subtle variations in the composition of the intra- and extracellular milieu. NO seems to play a part in various stages of carcinogenesis from initiation to progression. Expression of NOS have been detected in various human cancers. In breast cancer both the development of primary tumor and the process of metastasis seems to be influenced by the presence and amount of NO. In cervical carcinogenesis it acts as a molecular cofactor with HPV infection. Exhaled breath analysis and exhaled NO measurement may provide useful assays in providing diagnosis and disease progression in lung cancer. NO can initiate genetic alterations of gastric cells leading to gastric malignancy. Exposure of gastric epithelial cells to *H. pylori* bacterium may result in the generation of ROS and iNOS which in turn may cause genetic alterations leading to GC. Various studies have suggested a role of NO in the development of head and neck cancer. Thus NO seems to have an important part in the initiation, growth, and metastasis of various cancers. However, it is said to have a tumoricidal role as well. NO has been suggested to have a cytostatic and/or cytotoxic effect on tumor cells. However, this depends on various factors and once the cancer begins, NO seems to play protumoralrather than an antitumoral role. On the other hand, the tumoricidal properties of NO are being utilized in the treatment of cancer. NO can act as a novel potential therapeutic agent in patients with refractory cancer by sensitizing tumor cells to chemotherapy, radiotherapy or immunotherapy. Nevertheless, further validation and experimental/clinical trials are required to develop NO based strategies for cancer prevention and treatment.

## Abbreviations

CAG: Chronic atrophic gastritis; CSG: Chronic superficial gastritis; DYS: Dysplasia; GC: Gastric cancer; HNSCC: Head and neck squamous cell carcinoma; HPV: Human Papillomaviruses; IM: Intestinal metaplasia; MMP: Matrix Metalloproteinases; NO: Nitric oxide; NOS: Nitric oxide synthase; eNOS: Endothelial NOS; iNOS: Inducible NOS; QD: Quantum dots; RNS: Reactive nitrogen species; ROS: Reactive oxygen species; TIMP: Tissue inhibitors of MMP; VEGF: Vascular endothelial growth factor.

## Competing interests

The authors declared that they have no competing interests.

## Authors’ contributions

SK(C), designed, wrote and drafted the manuscript. MC, SB, ARG, and VJ participated in designing the manuscript and reviewed it. All authors have read and approved the final manuscript.
